# Supplementary material for: Bayesian Analysis of the Association between Casein Complex Haplotype Variants and Milk Yield, Composition, and Curve Shape Parameters in Murciano-Granadina Goats
Source: Animals (Basel). 2020 Oct 10;10(10):1845. doi: 10.3390/ani10101845 (PMC7600415; doi:10.3390/ani10101845)
Supplement: Supplementary file 1 [file animals-10-01845-s001.zip › Table S1.docx]

**Table S1:** Lactation curve model equations and where to find them in literature.

| **Model Name** | **Model regression equation** | **Reference** |
| --- | --- | --- |
| 1. Ali and Schaeffer (ALISCH) | $Y_{Days}= b_{0}+b_{1}Days+b_{2}Days+b_{3} \log\left( \frac{1}{Days} \right)+b_{4}\log\left( \frac{1}{Days} \right)2+e$ | Ali and Schaeffer,1987; Otwinowska-Mindur, 2015 and Strucken, 2011 |
| 1. Asymptotic Regression, Single Exponential decay to an arbitrary value (SXPDCY) | y=b_0_(1-b_1_)^Days^ | Garson, 2013 and Urbina, 2018 |
| 1. Asymptotic Regression,Lactation modification of Metcherlich Law of Diminishing Returns or Exponential growth model (METLAW) | $Y_{Days=}b_{0}\left( 1-b_{1}e^{{-b}_{2}Days} \right)-b_{3}Days+\varepsilon_{Days}$ | Prasad, 2007 |
| 1. Brody (BRODY) | $Y_{Days}= {b_{0}e}^{{-b}_{1}Days}- {b_{0}e}^{{-b}_{2}Days}$ | Brody et al, 1923; Sherchand, 1995; Duque, 2018 and Pulina, 2005 |
| 1. Cappio Borlino, biexponential (CAPBOR) | $Y_{Days}={b_{0}Days}^{b_{1}eb_{2}Days}$ | Cappio-Borlino et al., 1995; Martinez, 2018 and Franci, 1999 and Gipson, 1990 |
| 1. Cobby and Le Du (COBLDU) | $Y_{Days}=b_{0}(1-e^{-b2 Day})-b_{1}Days$ | Cobby and Le Du, 1978 and Duque, 2018 |
| 1. Compound/ Exponential Growth (CEXPGR) | $Y=b_{0}\left( {b_{1}}^{Days} \right)$ | Hobbie, 2007; Roberts, 2020 and Urbina, 2018 |
| 1. Cubic (CUBIC) | $Y_{Days}= b_{0}+ b_{1}Days+{b_{2}Days}^{2}+{b_{3}Days}^{3}$ | Mehta, 2015 |
| 1. Cubic Spline function with one knot (CUBSPL) | $Y_{Days}=b_{0}+b_{1}\left( Days \right)+b_{2}\left( Days \right)^{2}+b_{3}\left( Days \right)^{3}+b_{4}\left( Days-Knot \right)^{3}$  where Knot is treated as an additional parameter to be estimated, and represents the day of lactation where the knot-point occurs, i.e., where the three polynomial functions are linked. | [Verbyla & Verbyla, 2009](#_ENREF_40); [Cankaya et al., 2014](#_ENREF_8) and [Boyd, 2006](#_ENREF_7) |
| 1. Curve S (CURVES) | $Y=e^{\left( b_{0}+\left( \frac{b_{1}}{Days} \right) \right)}$ | Sikka, 1950; Mohanty, 2017; Biswal, 2017; Kokate, 2019; Cankaya, 2011; Zadeh, 2019; Brody et al, 1923; Bouallegue, 2019 and Korkmaz, 2011 |
| 1. Density (DENSITY) | $Y=\left( b_{0}+b_{1}Days \right)^{\frac{-1}{b_{2}}}$ | [Mellado et al., 2011](#_ENREF_26); [Nguyen et al., 2019](#_ENREF_29) and [Webster, 2018](#_ENREF_41) |
| 1. Dhanoa (DHANOA) | $Y_{\left( Days \right)}= {b_{0}Days}^{b_{1}b_{2}}e^{{-b}_{2Days}}$ | Dhanoa and Le Du, 1982 and Korkmaz, 2011 |
| 1. Dijkstra (DJKSTR) | $Y=b_{0}e^{b_{1}\left[ \frac{\left( 1-e^{{-b}_{2}Days} \right)}{b_{2}}-b_{3}Days \right]}$ | Dijkstra et al., 1997 and Nasri, 2008 |
| 1. Exponential decline function or Gaines (EDFGAIN) | $Y_{Days}=b_{0}e\left( -b_{2}Days \right)$ | Brody et al, 1923; Bouallegue, 2019 and Korkmaz, 2011 |
| 1. Gauss (GAUSS) | $Y=b_{0}e[-1/2 \left( (b_{1}- \mu)/\sigma\right)^{2}]$ | Guo, 2011 |
| 1. Gompertz (GMPRTZ) | $Y_{\left( Days \right)}={b_{0}e}^{{-b_{1}e}^{b_{2}}Days}$ | Bahashwan, 2018 and Nasri, 2008 |
| 1. Grossman (GROSMN) | $Y_{Days}=b_{0}Daysb_{1}e-b_{2}Days\left( 1+u sin\left( Days \right)+v cos\left( Days \right) \right)$ | Grossman et al., 1986 and Soysal, 2004 |
| 1. Hayashi (HAYSHI) | $Y_{Days}=b_{1}\left( exp-\frac{b_{2}}{Days}-exp-Days/b_{0}b_{2} \right)$ | Hayashi et al, 1986; [林孝, 1993](#_ENREF_45)and [Korkmaz et al., 2011](#_ENREF_20) |
| 1. Inverse quadratic polynomial (INVQPOL) | $Y_{Days}=Days\left( b_{0}+b_{1}Days+{b_{2}Days}^{2} \right)-1$ | Nelder, 1966; [Biswal et al., 2017](#_ENREF_5) and [Olori, 1997](#_ENREF_31) |
| 1. Inverse, linear Hyperbolic.(INVLINHY) | $Y_{Days}= b_{0}+\frac{b_{1}}{Days}$ | [Faro & Albuquerque, 2002](#_ENREF_13) |
| 1. Johnson Schumacher (JOHNSCH) | $Y= \frac{{b_{0}b_{1}}^{2}b_{2}}{\left( Days+ b_{1} \right)^{2}}\exp\left( \frac{b_{1Days}}{Days+b_{1}} \right)$ | [Ghavi Hossein-Zadeh, 2017](#_ENREF_16) |
| 1. Log Logistic (LOGLOG) | $Y_{Days}=b_{0}-ln\left( 1+b_{1}e^{-b_{2}Days} \right)$ | [Pina Pérez et al., 2007](#_ENREF_33) and [Bebbington et al., 2009b](#_ENREF_4) |
| 1. Log Modified Weibull (LGMWEIB) | $Y_{Days}=\left( b_{0}+ {(b}_{2}Days) \right)^{b_{1}}$ | [Carrasco et al., 2008](#_ENREF_9) |
| 1. Logarithmic (LOGARITH) | $Y_{Days}=lnb_{0}+b_{1}Days+{b_{2}Days}^{2}$ | [Dongre & Gandhi, 2013](#_ENREF_11) |
| 1. Madalena (MADALN) | $Y= b_{0}-b_{1}Days$ | Quinn, 2005; López, 2015; [Fresno Baquero et al., 1992](#_ENREF_15) and [Madalena et al., 1979](#_ENREF_24) |
| 1. Michaelis Menten (MICHMEN) | $\frac{b_{1}}{Days}\frac{1+\left( \frac{b_{2}}{210} \right)^{b_{1}}}{\left[ 1+\left( \frac{b_{2}}{Days} \right)^{b_{1}} \right]\left[ 1+\left( \frac{Days}{b_{2}} \right)^{b_{1}} \right]}$ | Rook et al., 1993 and [Rebouças et al., 2008](#_ENREF_34) |
| 1. MilkBot (MILKBOT) | $Y_{\left( Days \right)}=b_{0}\left( 1-\frac{e^{\frac{b_{2}Days}{b_{1}}}}{2} \right)e^{-b_{3}Days}$ | [Ehrlich, 2011](#_ENREF_12) |
| 1. Molina and Boschini/Modal Linear (MOL&BOS) | $Y= b_{0}-b_{1}\vert Days-b_{2}\vert$ | Quinn, 2005 and [Molina & Boschini, 1979](#_ENREF_28) |
| 1. Morgan Mercer Florin (MORMFLO) | $Y= \frac{b_{0}b_{1}+b_{2}{Days}^{b_{3}}}{b_{1}+{Days}^{b_{3}}}$ | López, 2015 |
| 1. Nelder, inverser polynomial, Yadav (NELDER) | $Y_{Days}=\frac{Days}{\left( b_{0}+b_{1}Days+{b_{2}Days}^{2} \right)}$ | Nanda, 2019 and Duque, 2018 |
| 1. Parabolic exponential model and Parabolic, Sikka (PEMSIK) | $Y_{Days}={b_{0}e}^{\left( b_{1}Days-{b_{2}Days}^{2} \right)}$ | Sikka, 1950; Mohanty, 2017; Biswal, 2017; Kokate, 2019; Cankaya, 2011 and Zadeh, 2019 |
| 1. Parabolic yield-density (PARYLDENS) | $Y=\left( b_{0}+b_{1}Days+b_{2}^{2}{Days}^{2} \right)^{-1}$ | [Yahuza, 2011](#_ENREF_42) |
| 1. Power (POWER) | $Y\left( Days \right)={b_{0}Days}^{b_{1}}$ | [Mellado et al., 2011](#_ENREF_26); [Nguyen et al., 2019](#_ENREF_29) and [Webster, 2018](#_ENREF_41) |
| 1. Quadratic cum log model (QDCMLOG) | $Y_{Days}=b_{0}+b_{1}Days+ {b2Days}^{2}+b_{3}ln\left( Days \right)+e$ | Malhotra et al., 1980; Biswal, 2017; [Lombaard, 2006](#_ENREF_23) and [Gupta et al., 2016](#_ENREF_17) |
| 1. Quadratic model (QUADRT) | $Y_{Days}=b_{0}+b_{1}Days+{b_{2}Days}^{2}$ | Biswal, 2017 [Noguera et al., 2011](#_ENREF_30) |
| 1. Quadratic model Dave (DAVE) | $Y_{Days}= b_{0}+b_{1}Days-{b_{2}Days}^{2}$ | Dave, 1971; [Bangar & Verma, 2017](#_ENREF_2) and Martinez, 2018 |
| 1. Quadratic spline function with one knot (QUADSPL) | $Y_{Days}=b_{0}+b_{1}\left( Days \right)+b_{2}\left( Days \right)^{2}+b_{3}\left( Days-Knot \right)^{2}$  where Knot is treated as an additional parameter to be estimated, and represents the day of lactation where the knot-point occurs, i.e., where the two polynomial functions are linked. | [Verbyla & Verbyla, 2009](#_ENREF_40); [Cankaya et al., 2014](#_ENREF_8) and [Boyd, 2006](#_ENREF_7) |
| 1. Ratio Cubics/ Partial Fraction with Cubic Denominator (RATCUB) | $\frac{\left( b_{0}+b_{1}Days+b_{2}{Days}^{2}+b_{3}{Days}^{3} \right)}{b_{4}{Days}^{3}}$ | [Kung, 2006](#_ENREF_21) |
| 1. Ratio Quadratics/ Partial Fraction with Quadratic Denominator (RATQUAD) | $\frac{\left( b_{0}+b_{1}Days+b_{2}{Days}^{2} \right)}{b_{3}{Days}^{2}}$ | [Kung, 2006](#_ENREF_21) |
| 1. Richards (RICHRDS) | $Y=b_{0}\left( 1-{b_{1}e}^{-kDays} \right)^{\frac{1}{b_{3}}}$ | Richards,1959; López, 2015 and Bayram, 2004 |
| 1. Rook (ROOK) | $Y=b_{0}\left[ \frac{1}{1+\left( \frac{b_{1}}{b_{2}+Days} \right)} \right]e^{b_{4}Days}$ | [Zadeh, 2019](#_ENREF_43) |
| 1. Simple Linear (SIMLIN) | $Y_{Days}= b_{0}+b_{1}Days$ | [Marsh, 1982](#_ENREF_25) and [Singh & Kumar, 2007](#_ENREF_38) |
| 1. Singh And Gopal (SIN&GOP) | $Y=b_{0}-b_{1}Days+b_{2} ln\left( Days \right)$ | Quinn, 2005; [Sherchand et al., 1995](#_ENREF_36); [Singh & Gopal, 1982](#_ENREF_39); [Bouallegue & M’Hamdi, 2019](#_ENREF_6) and [Noguera et al., 2011](#_ENREF_30) |
| 1. Third order Legendre ortogonal polynomial (3ORDLEG) | $Y=\beta_{0}P_{0}+\beta_{1}P_{1}+\beta_{2}P_{2}+\beta_{3}P_{3}$  $Y=b_{0}{0.7071\left( \left( 2\frac{\left( Days-1 \right)}{\left( 210-1 \right)} \right)-1 \right)}^{0}+b_{1}{1.2247\left( \left( 2\frac{\left( Days-1 \right)}{\left( 210-1 \right)} \right)-1 \right)}^{1}+b_{2}\left( -0.7906 \right)\left( \left( 2\frac{\left( Days-1 \right)}{\left( 210-1 \right)} \right)-1 \right)^{0}+{2.3717\left( \left( 2\frac{\left( Days-1 \right)}{\left( 210-1 \right)} \right)-1 \right)}^{2}+b_{3}{\left( -2.8062 \right)\left( \left( 2\frac{\left( Days-1 \right)}{\left( 210-1 \right)} \right)-1 \right)}^{1}+4.6771\left( \left( 2\frac{\left( Days-1 \right)}{\left( 210-1 \right)} \right)-1 \right)^{3}$  $P_{0}=0.7071w_{0}$; $P_{1}=1.2247w_{1}; P_{2}=-0.7906w_{0}+2.3717w_{2}$; $P_{3}=-2.8062w_{1}+4.6771w_{3}$; with w representing a standardized time unit calculated as  $w=\left( \frac{\left( 2\left( Days-1 \right) \right)}{\left( 210-1 \right)-1} \right)-1$ | [Schaeffer, 2004](#_ENREF_35); [León et al., 2012](#_ENREF_22) and [Boyd, 2006](#_ENREF_7) |
| 1. Verhulst/Logistic differential equation/Pearl Reed (VERHLST) | $Y=\frac{b_{0}}{\left( 1+{b_{1}e}^{\left( -b_{2}Days \right)} \right)}$ | [Herman & Strang, 2016](#_ENREF_18) and [Pearl & Reed, 1920](#_ENREF_32) |
| 1. Von Bertalanffy (VBRTLNFY) | $Y=b_{0}{(1-b_{1} e^{(-b2 Days)})}^{3}$ | Bahashwan, 2018;[Charruau, 2011](#_ENREF_10) and [Johansson & Hansson, 1940](#_ENREF_19) |
| 1. Weibull, Parametric Survival Models (PARSURW) | $Y_{Days}= b_{0}-b_{1}e^{-b_{2}{Days}^{b_{3}}}$ | Bebbington, 2009 ;[ZunZunSite3, 2020](#_ENREF_44);  [Bebbington et al., 2009a](#_ENREF_3) and [Franco García, 2016](#_ENREF_14) |
| 1. Wilmink’s exponential (WILMINK) | $Y_{Days}=b_{0}+{b_{1}e}^{-kDays}+b_{2}Days$  The only problem with it relies on the fact that the *k* parameter cannot be determined with linear regression and has therefore to be chosen beforehand. The original publication used a value of -0.05 for *k*. However, Silvestre et al. (2006) used a *k* of -0.065 that was found to be better in their study as also suggested by the better fit results obtained by other authors who applied it later. | Wilmink, 1987; [Silvestre et al., 2006](#_ENREF_37); [Mikhchi et al., 2015](#_ENREF_27) and [Arslan et al., 2004](#_ENREF_1) |
| 1. Wood (WOOD) | $Y_{Days}= {b_{0}Days}^{b_{1}}e^{{-b}_{2}Days}$ | Wood, 1967; Nasri, 2008; Gipson, 1990 and Bordonaro, 2013 |
